# Potential role of PIM1 inhibition in the treatment of SARS-CoV-2 infection

**DOI:** 10.1186/s43141-023-00520-x

**Published:** 2023-05-22

**Authors:** Magda M. F. Ismail, Rehab R. El-Awady, Amal M. Farrag, Sara H. Mahmoud, Noura M. Abo Shama, Ahmed Mostafa, Mohamed A. Ali, Mohammed H. Rashed, Iman H. Ibrahim

**Affiliations:** 1 Department of Pharmaceutical Chemistry, Faculty of Pharmacy (Girls), Al-Azhar University, Cairo, Egypt; 2 Department of Biochemistry and Molecular Biology, Faculty of Pharmacy (Girls), Al-Azhar University, Nasr City, 11884 Cairo Egypt; 3 Center of Scientific Excellence for Influenza Viruses, National Research Centre, Giza, Egypt; 4 Department of Clinical Pharmacy, Faculty of Pharmacy (Boys), Al-Azhar University, Cairo, Egypt

**Keywords:** *SARS-CoV-2*, *PIM1*, *PIM1 inhibitor*, *Notch pathway*, NRC-03-nhCoV

## Abstract

**Background:**

SARS-CoV-2 infection involves disturbing multiple molecular pathways related to immunity and cellular functions. PIM1 is a serine/threonine-protein kinase found to be involved in the pathogenesis of several viral infections. One PIM1 substrate, Myc, was reported to interact with TMPRSS2, which is crucial for SARS-CoV-2 cell entry. PIM1 inhibitors were reported to have antiviral activity through multiple mechanisms related to immunity and proliferation. This study aimed to evaluate the antiviral activity of *2-pyridone PIM1 inhibitor* against SARS-CoV-2 and its potential role in hindering the progression of COVID-19. It also aimed to assess PIM1 inhibitor’s effect on the expression of several genes of Notch signaling and Wnt pathways. In vitro study was conducted on Vero-E6 cells infected by SARS-CoV-2 “NRC-03-nhCoV” virus. Protein–protein interaction of the study genes was assessed to evaluate their relation to cell proliferation and immunity. The effect of *2-pyridone PIM1 inhibitor* treatment on viral load and mRNA expression of target genes was assessed at three time points.

**Results:**

Treatment with *2-pyridone PIM1 inhibitor* showed potential antiviral activity against SARS-CoV-2 (IC_50_ of 37.255 µg/ml), significantly lowering the viral load. Functional enrichments of the studied genes include negative regulation of growth rate, several biological processes involved in cell proliferation, and Interleukin-4 production, with interleukin-6 as a predicted functional partner. These results suggest an interplay between study genes with relation to cell proliferation and immunity. Following in vitro SARS-CoV-2 infection, Notch pathway genes, CTNNB1, SUMO1, and TDG, were found to be overexpressed compared to uninfected cells. Treatment with *2-pyridone PIM1 inhibitor* significantly lowers the expression levels of study genes, restoring Notch1 and BCL9 to the control level while decreasing Notch2 and CTNNB1 below control levels.

**Conclusion:**

*2-pyridone PIM1 inhibitor* could hinder cellular entry of SARS-CoV-2 and modulate several pathways implicated in immunity, suggesting a potential benefit in the development of anti-SARS-CoV-2 therapeutic approach.

**Supplementary Information:**

The online version contains supplementary material available at 10.1186/s43141-023-00520-x.

## Background

Coronavirus disease 2019 (COVID-19) is an emerging disease with significant morbidities and mortalities, caused by SARS-CoV-2 virus [1]. COVID-19 patients showed acute and long-term complications that could progress to serious illness especially in aged and immunocompromised patients [2].

SARS-CoV-2 entry routes were found to be through interaction of its spike protein (S-protein) with angiotensin-converting enzyme 2 (ACE2) receptors on cell membrane/transmembrane protease, Serine 2 (TMPRSS2). TMPRSS2 is responsible for proteolytic cleavage and activation of the S-protein of SARS-CoV-2, leading to fusion of the viral particles with cell membrane and viral entry [3]. Endosomal pathway is another route of SARS-CoV-2 entry via Cathepsin B, which is an endosomal cysteine protease. Cathepsin B releases the virus in cytoplasm through a mechanism similar to that of TMPRSS2 [3], although it works independently from TMPRSS2 [4]. Drugs targeting these routs could hinder viral entry and decrease viral load in infected cells.

Serine/threonine-protein kinase PIM-1 (PIM1) is an active serine/threonine kinase which plays a crucial role in cell survival via hindering apoptosis, among several biological processes [5]. PIM1 was also reported to be involved in host response to many viral infections, such as hepatitis B, Epstein-Barr, and human papilloma viral infections. Recently, it has been found that EV-A71 infection elevated both the mRNA and protein levels of PIM1. The elevated PIM promoted EV-A71 replication through enhancing IRES activity and blocking AUF1 cytosol translocation counteracting its antiviral property [6]. In addition, PIM1 was reported to promote Zika virus replication by suppressing host cells’ natural immunity through downregulating phosphorylation of both STAT1 and STAT2 which are essential for cellular antiviral response [7]. Hence, targeting PIM1 using PIM1 inhibitors could be effective in combating virus infection. The implication of PIM1 in immunosuppression during chronic viral infections was also reported. PIM1 was found to be highly expressed in suppressive neutrophils and had a crucial role in maintaining their survival and function. Thus, PIM1 inhibition mitigated suppressive neutrophils-mediated immunosuppression, with consequent increased CD8 T-cell function and improved viral control [8].

Previous studies had highlighted the interplay between PIM1 and Myc, which is a transcription factor known to be a PIM1 substrate [9]. Several studies had pointed out some interaction between Myc and TMPRSS2 [10, 11]. However, on a mechanistic level, the interaction of PIM1 and its substrate, Myc, with TMPRSS2 is not clear yet.

Notch signaling has been reported to facilitate the infectivity of many viruses including Epstein-Barr virus, the human papillomavirus (HPV), hepatitis B virus (HBV), and hepatitis C virus (HCV). In SARS-CoV-2, it was found that Notch1 can indirectly enhance viral entry through inducing FURIN expression, the protease responsible for exposing the fusion sequences of the viral S-protein, facilitating viral particle fusion with cell membrane [12]. Furthermore, the implication of the Notch pathway and downstream genes in many COVID-19 events as cytokines storm, hypoxic response, and coagulopathic response has been reported [13].

The interaction between PIM kinases and Notch signaling pathways has been studied. It was reported that PIM kinases induced Notch1 activity via phosphorylation, promoting its signaling along with other signaling pathways such as the Wnt pathway [14]. PIM1 inhibitors could modulate molecular functions of PIM1. One of them is a 2-pyridone compound *(6-amino-4-(3,4-dimethoxyphenyl)-1-(2-ethylphenyl)-2-oxo-1,2-dihydropyridine-3,5-dicarbonitrile)*, named *2-pyridone PIM1 inhibitor* hereafter, had shown significant PIM1 inhibition with minor toxicity risk in vitro [15].

This study aimed to evaluate the antiviral activity of *2-pyridone PIM1 inhibitor* against SARS-CoV-2 and its potential role in hindering the progression of COVID-19. It also aims to assess PIM1 inhibitor’s effect on Notch signaling pathway, Wnt pathway, and some key genes involved in SARS-CoV-2 pathogenesis.

## Methods

### *2-Pyridone PIM1 inhibitor synthesis and preparation*

It is described previously [15]*.*

### Cytotoxicity and antiviral activity

Vero-E6 cells were sustained in DMEM (Dulbecco’s Modified Eagle’s Medium) containing 1% penicillin/streptomycin mixture and 10% fetal bovine serum (FBS) in a humidified incubator at 37 °C and 5% CO_2_. SARS-CoV-2 (NRC-03-nhCoV) virus [16] was propagated and titrated as described previously [17].

### ***Cytotoxicity (CC***_***50***_***) assay***

Half-maximal cytotoxic concentration (CC_50_) of *2-pyridone PIM1 inhibitor* was assessed in VERO-E6 cells using crystal violet as described previously [18]. Briefly, cells were seeded in 96-well plates as 100 µL/well with a density of 3 × 10^5^ cells/ml. Plates were incubated for 24 h in a 5% CO_2_ humidified incubator at 37 °C. After the incubation period, cells were treated with various concentrations of the compound in triplicates. After 72 h, the supernatant was discarded, and cell monolayers were fixed (using 10% formaldehyde for 1 h at room temperature), dried, and stained with crystal violet on a bench rocker (50 µl of 0.1% solution, for 20 min at room temperature). After washing and drying the stained cell monolayers, methanol (200 µl/well for 20 min) was used to dissolve the crystal violet on a bench rocker at room temperature. Crystal violet solution absorbance was measured at *λ*max 570 nm using a plate reader. The CC_50_ of *2-pyridone PIM1 inhibitor* was calculated as the concentration required to induce cytotoxicity by 50% in the treated cells relative to the virus control.

### Inhibitory concentration _50_ (IC_50_) determination

The IC_50_ value for *2-pyridone PIM1 inhibitor* was determined as described previously [17], with slight modifications. Briefly, VERO-E6 cells were seeded in tissue culture plates (2.4 × 10^4^/well) and incubated overnight in a humidified incubator at 37 °C, 5% CO_2_. The cell monolayers were then washed using 1 × PBS. An aliquot of the SARS-CoV-2 virus containing 100 TCID_50_ was incubated with serial dilutions of *2-pyridone PIM1 inhibitor* and kept at 37 °C for 1 h. The VERO-E6 cells were treated with the virus alone or virus/*2-pyridone PIM1 inhibitor* and incubated at 37 °C in a total volume of 200 µl per well along with uninfected untreated cells as a control and untreated infected cells as a virus control. After incubation in a 5% CO_2_ incubator for 72 h at 37 °C, cells were fixed using 10% paraformaldehyde for 20 min and stained with 0.5% crystal violet in dH_2_O for 15 min at room temperature. Absolute methanol (100 μl/well) was used to dissolve crystal violet. Crystal violet solution absorbance was measured at *λ*max 570 nm using a plate reader. The IC_50_ of the compound is the concentration required to reduce the virus-induced cytopathic effect (CPE) by 50%, relative to the virus control.

### Protein–protein interaction prediction

GeneMANIA prediction tool [19] and STRING [20] were used to predict the interaction between the study genes and to evaluate the protein–protein interaction (PPI) enrichment. VirusMINT (http://mint.bio.uniroma2.it/virusmint/RRID:SCR_005987) was used for multivariate interaction between host and viral proteins. Gene list input in STRING and VirusMINT was PIM1, Notch1, Notch2, CTNNB1, CDC42, PSEN1, BCL9, ACTR2, TDG, SUMO1, CDC34, and IL-6. *p*-value < 0.05 and false discovery rate (FDR) < 0.05 were considered significant.

### Gene expression assay

#### Cell treatment

Five-hundred TCID50 of SARS-CoV-2 were used to infect VERO E6 cells. Cells with and without treatment with *2-pyridone PIM1 inhibitor* were collected in 1 × PBS pellets by centrifugation at time intervals of 6, 12, and 24 h post-infection. Control cells are uninfected untreated cells.

#### qPCR

Total RNA was extracted from treated and untreated cells (at three time points: 6, 12, and 24 h post-infection) using RNeasy Mini Kit, following the manufacturer’s instructions. The extracted mRNA was reverse-transcribed into cDNA using RevertAid First-Strand cDNA Synthesis Kit (Thermo Scientific USA). Primers were designed for relative quantification of the study genes (Notch1, 2, CTNNB1, PSEN1, CDC42, BCL9, ACTR2, SUMO1, and TDG), using Primer3 software (https://primer3.ut.ee/, RRID:SCR_003139). SYBR Green qPCR Master Mix (Thermo Scientific, USA) was used to prepare the reaction mixture with GAPDH as a housekeeping gene. Technical replicates were prepared for the three time point samples (6, 12, and 24 h post-infection). Results were expressed using the 2^−ΔΔCt^ method. Primers and annealing temperature *T*_a_ for each are listed in Table 1. qPCR cycling conditions were 95 °C for 10 min followed by 40 cycles (95 °C for 15 s, *T*_a_ for 30 s, and 72 °C for 40 s).

### Statistical analysis

Two-tailed independent Student’s *t*-test for parametric data was used to assess the significant differences between the values of the control and treatment groups where *p*-value < 0.05 was considered significant. Experiments and assays were performed in biological and technical replicates.

## Results

### Antiviral activity

**Fig. 1:**
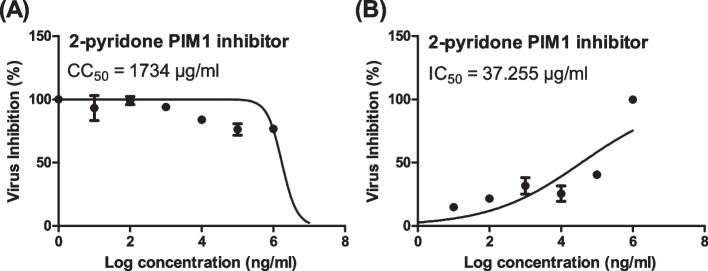
Cytotoxicity and in vitro anti-SARS-CoV-2 activity of **A** and **B**, respectively. **A** Cytotoxicity of 2-pyridone in Vero E6 cells as measured by MTT assay. **B** Dose-inhibition curves against SARS-CoV-2 “NRC-03-nhCoV” virus in Vero E6 cells. Cytotoxic concentration 50 (CC_50_) and inhibitory concentration 50 (IC_50_) values were calculated using nonlinear regression analysis of GraphPad Prism software (version 5.01) by plotting log inhibitor versus normalized response (variable slope)

The CC_50_ of *2-pyridone PIM1 inhibitor* was 1734 µg/ml that showed a potential antiviral activity against SARS-CoV-2 with an IC_50_ of 37.255 µg/ml (Fig. 1). SARS-CoV-2 viral load in VERO-E6 cells treated with *2-pyridone PIM1 inhibitor* was found to be considerably low compared to non-treated SARS-CoV-2-infected VERO-E6 cells.

### Protein–protein interaction prediction

**Fig. 2:**
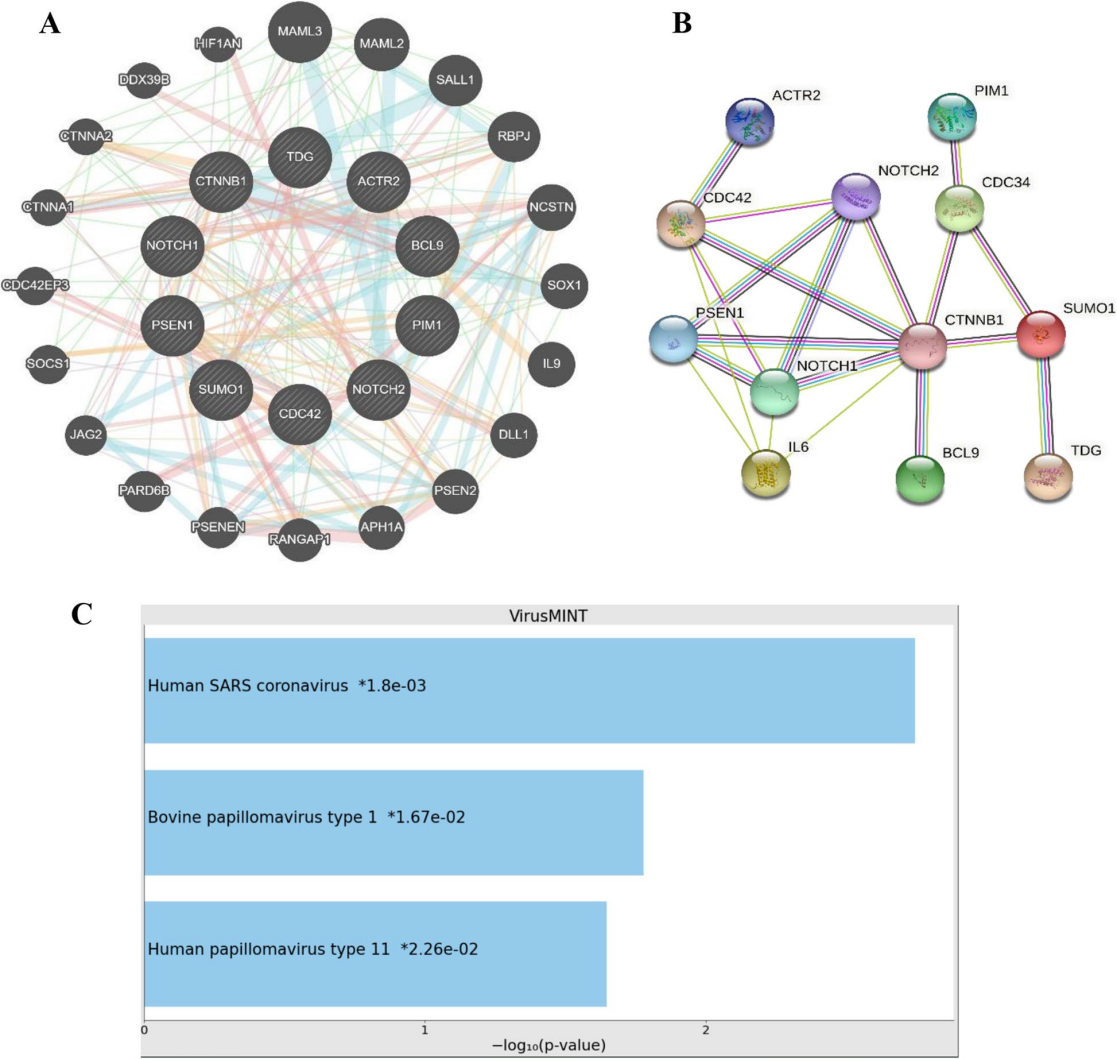
Protein–protein interaction and functional prediction. **A** Protein–protein interaction network of PIM-1 with the study genes, showing prominent physical interaction (dark pink lines), pathways (light blue lines), and co-expression (violet lines). **B** Protein functional enrichment network of the study genes. **C** Significant interactions of the study genes with viral proteins, assessed through VirusMINT database

Types of interactions between studied genes are displayed in Fig. 2A, showing physical interaction (47.15%) between the study genes and several genes involved in immunity including IL-6 and multiple pathway interactions. PIM1 was shown to have a significant genetic interaction with BCL9 (*p*-value: 0.0004), ACTR2 (*p*-value: 0.0007), and CDC42 effector protein (*p*-value: 0.0006) along with co-expression with PSEN1 (*p*-value: 0.0153) and TDG (*p*-value: 0.016) (supplementary file 1).

Functional enrichments of the studied genes (network PPI enrichment *p*-value: 0.00134, Fig. 2B) include the following:

1. Negative regulation of growth rate (GO: 0,045,967/FDR 0.00091)
2. Several biological processes involved in cardioblast proliferation and heart development (GO: 0,003,264/FDR 0.00016, GO: 0,003,266/FDR0.0027, GO: 0,003,161/FDR 0.0041, GO: 0,061,311/FDR 0.00043, and GO: 0,061,314/FDR 0.0009)
3. Positive regulation of neuroblast, astrocytes, and glial cells proliferation (GO: 0,002,052/FDR 0.0084, GO: 0,048,710/FDR 0.0104, GO: 0,060,251/FDR0.0119)
4. Interleukin-4 production (GO:0,032,633/FDR 0.0027)

Protein network of the study genes showed that the predicted functional partners are interleukin-6; (IL-6) and ubiquitin-conjugating enzyme E2 R1 (CDC34) (Fig. 2B, Supplementary file 2).

Multivariate interactions prediction of the study proteins with viral proteins showed significant interaction between the studied protein list and SARS-CoV proteins (Fig. 2C).

### Effect of SARS-Cov-2 and 2-pyridone PIM1 inhibitor on some Notch signaling and Notch downstream genes

**Fig. 3:**
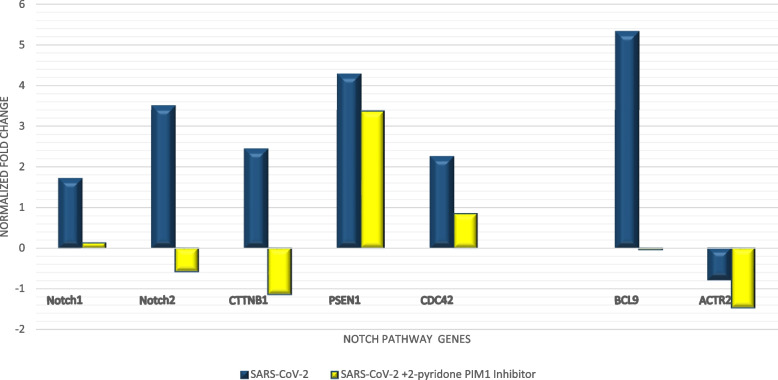
Expression of Notch signaling and downstream genes after 24 h of SARS-CoV-2 infection in vitro. Expression levels are shown relative to control, with and without *2-pyridone PIM1 inhibitor* treatment

SARS-CoV-2 infection resulted in a significant overexpression of Notch1, Notch2, CTNNB1, CDC42, and PSEN1 after 24 h of infection. No significant change in expression levels of all genes was observed at time points 6 and 12 h post-infection, compared to control. Treatment with *2-pyridone PIM1 inhibitor* was able to significantly downregulate Notch1, Notch2, CTNNB1, and CDC42 in SARS-CoV-2-infected cells at 24 h post-infection (*p*-value < 0.001). The expression of Notch1 was normalized, while the expression levels of Notch2 and CTNNB1 were below those of the control cells. The expression levels of CDC42, although significantly decreased, remained above those of the control cells. On the other hand, the change in PSEN1 expression levels was insignificant after 2-pyridone PIM1 inhibitor treatment compared to untreated infected cells (Fig. 3).

The expression levels of BCL9 and ACTR2, which are Notch-signaling downstream genes, were assessed. SARS-CoV-2 infection caused overexpression of BCL9 and underexpression of ACTR2 after 24 h of infection of the cells. *2-pyridone PIM1 inhibitor*-treated cells showed significantly lower levels of both genes (*p*-value < 0.0001). BCL9 levels were restored to levels close to those observed in control cells, while ACTR2 levels were lower in treated cells compared to both control cells and infected untreated cells (Fig. 3).

### Effect of SARS-CoV-2 and 2-pyridone PIM1 inhibitor on TDG and SUMO 1

SARS-CoV-2 infection resulted in a significant overexpression of SUMO1 and TDG after 24 h but insignificant compared to control cells at 6 h post-infection. At the 12-h time point post-infection, TDG expression showed significant overexpression compared to control cells, while SUMO1 remained unchanged.

**Fig. 4:**
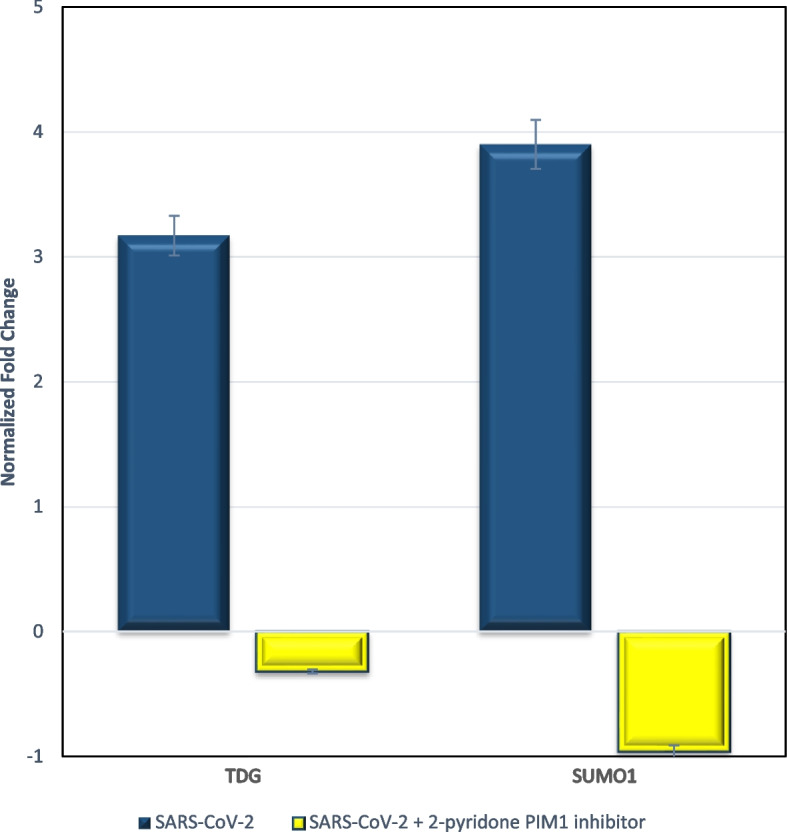
Expression of TDG and SUMO1 after 24 h of SARS-CoV-2 infection in vitro. Expression levels are shown relative to control, with and without *2-pyridone PIM1 inhibitor* treatment

Treatment of SARS-CoV-2-infected cells with *2-pyridone PIM1 inhibitor* caused significant downregulation of TDG and SUMO1 expressions below expression levels in control cells (*p*-value < 0.0001) compared to untreated SARS-CoV-2-infected cells (Fig. 4).

## Discussion

The development of specific antiviral agents for SARS-CoV-2 is still a major concern of many studies. Immunomodulation, protease inhibition, and targeting the viral RNA-dependent RNA polymerase (RdRp) are potential targets for anti-coronavirus agents [21]. PPI functional enrichment prediction of the present study genes showed significant enrichment of genes related to immunity, such as interleukin-4 production, with IL-6 and CDC34 as predicted functional partners. Notch1 was previously reported to positively regulate IL-6 production in macrophages, which in turn amplifies the Notch signaling leading to further production of IL-6 — through positive feedback mechanism. Also, Notch signaling promotes the production of inflammatory Th1/Th17 cytokines in T-helper cells [12].

PPI functional enrichment prediction suggested a role of the study genes in growth and proliferation of different types of cells including cardiac and CNS cells. However, the role of these genes in cardiac and neuronal complications of COVID-19 and long COVID needs further investigations. Host–pathogen interaction analysis by VirusMINT database showed a significant interaction between coronaviruses and the current study genes. These results suggest that the interplay between the study genes and SARS-CoV-2 might help in evaluating new treatment approaches Table 1.

**Table 1:** Primer sequences of target genes

	**Primer sequence (5′-3′)**		***T***_**a**_
	**Forward**	**Reverse**	
**Notch1**	ATGTGTTCTCGGAGTGTGTATG	AGGGACCAAGAACTTGTATAACC	55 °C
**Notch2**	CAGGTGAATTCCCGACTCTTT	ACCGACAGACAAATCAGGTAAG	55 °C
**CTNNB1**	CTTCACCTGACAGATCCAAGTC	CCTTCCATCCCTTCCTGTTTAG	55 °C
**PSEN1**	CGGGATTCCCATTCTGTAGTC	CTGTCTGAGGCCACGTAAAT	54 °C
**CDC42**	GATGTAAGCAGGCAGAGGTAAG	GGCACAGGCACACAGAATA	55 °C
**BCL9**	CTGGGAAATGTAGAGTCAGGTG	GCTCTGGAGGCATGGTATAAG	55 °C
**ACTR2**	CTCACAGAACCTCCTATGAACC	CTGCCTGGATGGCTACATATAC	55 °C
**SUMO1**	CCCTTCATATTACCCTCTCCTTT	CACTTGCATTGGTCGATCTTATT	54 °C
**TDG**	AGCCACGAATAGCAGTGTTTA	CTGAAGCCCAAATTCCAAGTTC	55 °C

PIM1 kinase was reported to be highly expressed in the bronchial epithelium. Inhibition of PIM1 kinase was shown to reduce viral replication and viral particles release in cultured PBECs through induction of apoptosis during viral infection [5]. The present study provided evidence of the role of PIM1 inhibition in lowering the viral load. SARS-CoV-2- infected cells treated with *2-pyridone PIM1 inhibitor* showed lower viral load compared to untreated infected cells, possibly through the effect of PIM1 inhibition on TMPRSS2 through the PIM1 kinase substrate, Myc. However, understanding the mechanism of this effect needs further investigation.

The initial viral load is not necessarily associated with the severity of symptoms or duration of disease [22]. Hence, hindering viral entry is not sufficient to prevent COVID-19 progression. Studying the effect of *2-pyridone PIM1 inhibitor* on pathways implicated in immunity could provide a better idea about its therapeutic effect and mechanism of action.

To assess the effect of PIM1 inhibition on the molecular pathogenesis of SARS-CoV-2, expression of some Notch pathway genes, Wnt/β-catenin signaling pathway, TDG, and SUMOylation, was studied in the current study.

The significant overexpression of Notch1 induced by SARS-CoV-2 infection was normalized in cells treated with *2-pyridone PIM1 inhibitor*. For Notch2, the significant overexpression induced by SARS-CoV-2 infection was reduced in cells with *2-pyridone PIM1 inhibitor* below the control levels.

Notch pathway has a crucial role in the activation and the differentiation of innate and adaptive immune cells [12].

In SARS-Cov2 infection, a previous study using a computational model reported that the proteins interacting with SARS-CoV-2 RNA 5′-region were associated with Notch2 receptor signaling [23]. In addition, Notch signaling indirectly promotes viral entry through Furin induction by Notch1 [12]. Furthermore, the involvement of Notch signaling in cytokines storm, coagulopathy, and hypoxic events accompanied SARS-CoV-2 infection has been reported [13].

Hence, the ability of 2*-pyridone PIM1 inhibitor* to normalize the expression of Notch 1 and reduce the expression of Notch 2 below the control levels could provide a mechanism by which *2-pyridone PIM1 inhibitor* could be effective in treatment of COVID-19. Previous studies reported that inhibition of Notch signaling pathway can hinder SARS-CoV-2 infection, decrease inflammatory responses, and help alveolar regeneration [24].

Another pathway implicated in host–pathogen interaction is the Wnt/β-catenin signaling pathway. Several previous studies reported the crucial role of Wnt/β-catenin signaling pathway in many viral infections including *Flavivirus* [25], influenza virus [26], and herpes simplex virus 1 [27]. All those studies reported that the activation of Wnt/β-catenin signaling during viral infection disturbs cellular homeostasis and provides a pathogenic environment that induces viral replication. Also, they reported that inhibition of Wnt/β-catenin signaling repressed viral replication and had strong antiviral effect.

In the present study, CTNNB1 was overexpressed after SARS-CoV-2 infection, in agreement with previous studies [28, 29]. ِAfter *2-pyridone PIM1 inhibitor* treatment, CTNNB1 expression was significantly decreased, even below its levels in control cells. The explanation and potential effects of this downregulation need further studies. During SARS-CoV-2 infection, Wnt/*β*-catenin pathway was reported to be upregulated in association with TGF-b and STAT pathway leading to stimulate immune signaling, increase inflammatory cytokines production, and promote pulmonary fibrosis. Thus, Wnt/*β*-catenin signaling inhibition could negatively modulate SARS-CoV-2 infection [28].

Being one of the Rho family GTPases, CDC42 plays a vital role in the regulation of actin cytoskeleton, vesicle trafficking, and cell–cell adhesion. Thus, several viruses including Ebola virus, respiratory syncytial virus, and coronavirus hijack CDC42 to facilitate viral cell entry and subsequent nuclear infiltration [30]. Also, Kolyvushko et al. (2020) identified that the activation of CDC42 by EHV-1 is required for stabilization of tubulin to promote intracellular virus transport and cell-to-cell virus spread. They also found that the chemical inhibition of CDC42 resulted in precluding virus trafficking to the nucleus of infected cells [31]. In the current study, CDC42 was upregulated in SARS-CoV-2-infected cells. Treatment with *2-pyridone PIM1 inhibitor* was able to downregulate its level, yet it remained higher than the control levels. In line with our results, a previous study showed alteration in expression of CDC42 in nasopharyngeal swabs of COVID‐19 compared with non‐COVID‐19 patients. Also, they found downregulation of CDC42 expression upon treatment of human lung carcinoma A549 cells with Rho GTPases inhibitor as atorvastatin [32]. The role of CDC42 in COVID-19 progression remains unclear. However, an in vitro study performed on Vero E6 cells confirmed that SARS-CoV-2 depended on CDC42 as one of different signaling pathways involved in viral cell entry and cell–cell fusion mechanisms [33].

In the current study, Notch signaling downstream genes, BCL9 and ACTR2, were also assessed. SARS-CoV-2 infection caused overexpression of BCL9 and underexpression of ACTR2 in infected cells after 24 h post-infection. Treatment with *2-pyridone PIM1 inhibitor* was able to restore BCL9 expression to a level near that of control cells, while the level of ACTR2 expression was lower in the treated cells than both in control cells and infected untreated cells. Previously, a strong interaction between BCL9 and the Wnt/*β*-catenin pathway was reported. BCL9 acts as a transcriptional coactivator of CTNNB1 protein via binding to its N-terminus [34]. Thus, BCL9 knockdown strongly inhibits the transcriptional activity of CTNNB1 and also the expression of various downstream genes in the Wnt/*β*-catenin pathway [35].

ACTR2 gene codes for ARP2 protein which has a vital role in actin polymerization, cell shape, and motility. Also, ARP2 has a crucial role in RSV spread through formation of filopodia [36]. Recently, it was suggested that ARP2 plays a role in host–pathogen interaction in SARS-CoV-2 infection. Moreover, inhibition of viral RNA expression was reported upon pharmacological inhibition of ARP2/3 complex [37]. Collectively, the studied Notch pathway genes were increased with increased viral load after SARS-CoV-2 infection, with the exception of ACTR2 as discussed above. Treatment with 2-pyridone PIM1 inhibitor decreased the viral load and was able to significantly reverse the effect of SARS-CoV-2 infection on the Notch pathway genes. As detailed above, Notch pathway was previously shown to interact with many pathogens including SARS-CoV-2, modulating their infectivity and viral entry [13]. Hence, results of the current study suggest that the 2-pyridone PIM1 inhibitor could hinder SARS-CoV-2 infectivity via modulating its interaction with Notch pathway.

In the present study, SARS-CoV-2-infected cells showed a significant overexpression of SUMO1 and TDG. Treatment with *2-pyridone PIM1 inhibitor* significantly downregulated both genes to levels even below their levels in control cells.

SUMOylation pathway was reported to have a potential role in regulating cell homeostasis during viral infection. Various viruses were found to be able to hijack host cells’ SUMO pathways to promote virus replication and pathogenesis [38]. Also, SUMOylation was reported to play a vital role in regulating innate immunity through altering interferon production and inhibiting NF-κB transcription [39].

As the *2-pyridone PIM1 inhibitor* significantly shifted SUMO1 levels below control levels, inhibition of SUMO1 could be a part of the *2-pyridone PIM1 inhibitor* antiviral activity. As reported by [38], alteration in expression of genes regulating SUMO pathways was found in COVID-19 patients, suggesting SUMO pathway as a target for anti-coronavirus therapy. In addition, the interaction between the N-protein of SARS-CoV and SUMO machinery was documented. Thus, the interaction between SARS-CoV-2 and SUMO is possible due to significant similarity of the N-proteins among coronaviruses [40].

SUMOylation was reported to affect the substrate binding capacity and catalytic activity of TDG enzyme [41]. TDG was found to be upregulated in the current study in SARS-CoV-2-infected cells. *2-pyridone PIM1 inhibitor* decreased its levels below the control levels. The ability of SUMO1 to regulate TDG activity could explain the TDG overexpression after SARS-CoV-2 infection [42]. Another study revealed that human TDG could be manipulated by SARS-CoV2 during the genome organization leading to the appearance of mutated strains with subsequent increase in the viral transmission rate and pathogenicity [43].

## Conclusion

*Treatment with 2-pyridone PIM1 inhibitor* could hinder SARS-CoV-2 cell entry and lower the viral load. In addition, *2-pyridone PIM1 inhibitor* could significantly lower the expression levels of Notch pathway genes, CTNNB1, SUMO1, and TDG, which were overexpressed by SARS-CoV-2 infection. Thus, *2-pyridone PIM1 inhibitor* could affect SARS-CoV-2 pathogenesis through modulation of several pathways implicated in immunity, suggesting its potential use in the development of anti-SARS-CoV-2 therapeutic approach.

## Supplementary Information

**Additional file 1:** PPI prediction between PIM1 and the studied genes.

**Additional file 2:** Functional PPI enrichments of the studied genes.

## Data Availability

All data generated or analyzed during this study are included in this published article and its supplementary information files.

## Abbreviations

COVID-19: Coronavirus disease 2019
S-protein: Spike protein
ACE2: Angiotensin-converting enzyme 2
TMPRSS2: Transmembrane protease, serine 2
PIM1: Serine/threonine-protein kinase pim-1
HPV: Human papillomavirus
HBV: Hepatitis B virus
HCV: Hepatitis C virus
DMEM: Dulbecco’s Modified Eagle’s Medium
FBS: Fetal bovine serum
CC_50_: Half-maximal cytotoxic concentration
IC_50_: Inhibitory concentration
CPE: Cytopathic effect
PPI: Protein–protein interaction
FDR: False discovery rate
IL-6: Interleukin-6
